# Refer-To-Pharmacy: Pharmacy for the Next Generation Now! A Short Communication for *Pharmacy*

**DOI:** 10.3390/pharmacy3040364

**Published:** 2015-12-11

**Authors:** Alistair Gray

**Affiliations:** Clinical Services Lead Pharmacist, East Lancashire Hospitals NHS Trust, Royal Blackburn Hospital, Haslingden Road, Blackburn, Lancashire BB2 3HH, UK; E-Mail: alistair.gray@elht.nhs.uk; Tel.: +44-1254-732-252; Fax: +44-1254-736-850

**Keywords:** Medicines optimisation, transfer of care, referral, hospital pharmacy, community pharmacy, new medicine service, medicines use review, medicines waste, re-admission, medicines adherence

## Abstract

Refer-to-Pharmacy is the first fully integrated hospital to community pharmacy referral system. This article explains the importance of these referrals for patients and health economies to improve medicines optimisation, and how Refer-to-Pharmacy works in both hospital and community pharmacies.

## 1. Introduction

There has been considerable change in pharmacy within the space of a generation. Time was you either went down the hospital or community route and never the twain shall meet. Community pharmacy was all about dispensing; good service perceived as how quickly medicines could be dispensed…with assumed accuracy. Hospital pharmacy was little different, technicians were called “dispensers” and pharmacists were just venturing on to wards; even the prescription charts were in black and white! Now, the world has moved on; dispensing still happens, often by robots, while community pharmacists run Healthy Living Pharmacies [1] delivering health interventions and adherence consultations; and teams of hospital pharmacists and technicians are part of multi-disciplinary teams working on wards and in clinics where electronic prescribing is the norm.

Finally, the twain have met; there has never been a better time for hospital and community pharmacists to work together for the benefit of patients and the health economy at large. The main drivers for this change have been the introduction of new community pharmacy services, tied in with a greater desire from the pharmacy profession to improve transfer of care and for pharmacists to have access to relevant clinical information, historically the domain of family doctors. In the National Health Service (NHS) in England there is the New Medicine Service (NMS) [2] and targeted Medicines Use Reviews (MUR) [3] including post-discharge MURs; in NHS Wales there is the Discharge Medication Review (DMR).

Elliott *et al* have shown the NMS produces a 10% improvement in medicines adherence [4], which means more people taking their medicines as intended and gaining health benefits. Hodson *et al* have shown DMRs produce a three-fold return on investment through less wasted medicines and fewer episodes of unscheduled care [5], again leading to more people taking their medicines correctly and staying healthy at home. 

What has historically been extremely difficult is finding a way to “target” referable patients at their community pharmacist in a timely manner, provide the community pharmacist with the right information to make an intervention, and doing so consistently so *every* eligible patient is referred.

## 2. Options for Hospital to Community Pharmacy Referral

At East Lancashire Hospitals NHS Trust it was originally thought signposting patients to their community pharmacist and including a referral note in their discharge letter would suffice, but like colleagues who tried this approach in other parts of the country [6], this has proved to be an impractical strategy; patients do not follow through. There are some centres that use faxed or phoned through referrals, but these are very time consuming, and there are information governance risks using faxes; in practice this is an unfeasible option for making large numbers of referrals. In Newcastle hospitals an electronic form in PharmOutcomes is used to inform community pharmacists of patients eligible for various kinds of post-discharge intervention [7]. A referral only takes place at the point of discharge with patient demography entered manually and selected elements of the discharge prescription manually copied and pasted in.

To be truly effective, a referral solution should be electronic, integrated with hospital IT systems to allow auto-population of patient demographics, provide a copy of the patient’s discharge letter, be quick and easy to use at both the hospital and community ends, and be as automated as possible to make it feasible to routinely refer *every* eligible patient. Refer-to-Pharmacy was conceived and developed to meet this ethos in a joint venture between East Lancashire Hospitals NHS Trust and software developers Webstar-Health. Furthermore it is designed to be easily integrated with IT systems in other health economies making diffusion and adoption of the innovation easy.

## 3. Refer-to-Pharmacy—How It Works in Hospital

Refer-to-Pharmacy works in tandem with pharmacists and pharmacy technicians performing their professional practice on wards, where they routinely identify patients who are eligible for an NMS or targeted MURs in the course of screening prescriptions or participating in ward rounds. Patients may even be identified on admission as part of medicines reconciliation; referrals are not just limited to consultation-referrals. Care home residents or blister pack users have an information-referral sent informing their community pharmacist initially that they are in hospital (and to pause any dispensing activity for that patient), with a later second message sent *automatically* at discharge informing them of any changes to medicines. This means fewer medicines are unnecessarily dispensed, saving time and the cost of wasted medicines; and the community pharmacist can act as a safety net next time a prescription arrives from the patient’s family doctor to ensure that changes made in hospital are reflected on their latest prescription.

Referral routes can be easily added depending on local services e.g., in East Lancashire there is a “home visit” option to refer patients to the domiciliary medicines support team, where vulnerable patients get extra help from a pharmacist or technician in their own home.

For NMS and MUR consultation-referrals, it is important that patients understand why being referred to their community pharmacist matters. To help explain this, patients are shown a short film on their bedside TV before being consented to a referral. The film explains the problems of poor adherence in lay language, what has been done to help, and what benefits they can expect to gain from a community pharmacy consultation. The film delivers these messages in a consistent, positive way, and is designed as an advert for community pharmacy enhanced services so that patients willingly consent to referral. This film can be viewed at the Refer-to-Pharmacy website [8] along with other useful resources about Refer-to-Pharmacy, including a short screen capture film showing a referral being made in real time.

If a patient consents to a consultation or an information-referral, the pharmacist or technician signs into the Refer-to-Pharmacy application on their preferred IT device. This also means the referrers contact details auto-populate the referral so any later queries can be directed to the right person.

It is then possible to make a referral with little or no typing. If a mobile device, such as a tablet computer with a scanner is used, the patient’s identifier barcode can be scanned to input their hospital number; else it is typed in. Refer-to-Pharmacy instantly draws down the patient’s demography (name, address, date of birth, contact number, NHS number, GP contact details) and any previous referral history.

**Figure 1:**
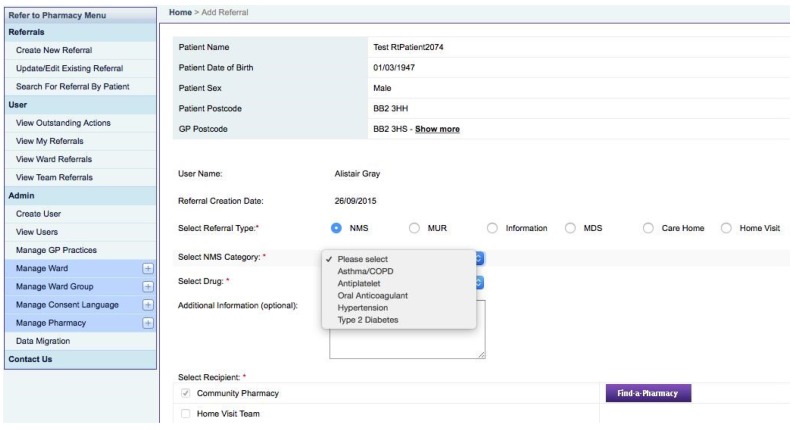
Refer-to-Pharmacy: referral selection screen.

Consent is sought, gained, and formally acknowledged electronically, with the displayed consent statement being available in a variety of languages. A series of option buttons then appear to select the desired referral route: *NMS, MUR, Information only, Care Home, MDS, Home Visit*. If NMS or MUR is selected, various dropdown options appear allowing precise selection of the type of NMS or MUR required, and ensuring that the patient is eligible for current commissioned services (Figure 1). A Home Visit referral opens up an additional electronic-form that uses pick lists to select the extra information the domiciliary pharmacy team need. There is an optional free text box that can be completed for any referral type should this be required.

**Figure 2:**
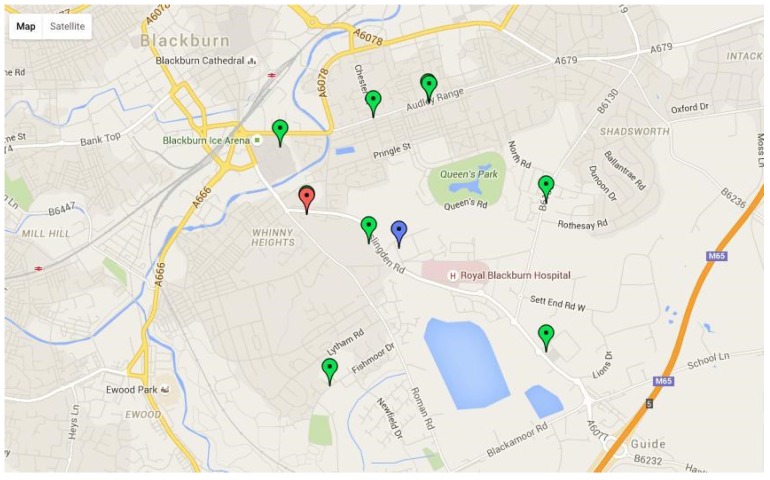
Refer-to-Pharmacy: “Find-a-Pharmacy” interactive map.

The final stage for community pharmacy referrals is Find-a-Pharmacy. Patients may know exactly which pharmacy they use, or have a labeled pack with a printed address, which can be used to select their pharmacy from an interactive list. Alternatively there is an interactive map to help a patient navigate to their pharmacy—green pins are pharmacies, the blue pin is the patient’s address, the red pin their GP. (Figure 2).

It takes seconds to complete the referral process making it easily absorbable into daily routines *and* feasible to refer *every* eligible patient on each ward. Referrals can be made at *any* point in the patient’s hospital journey, right from admission until discharge. Information-referrals have an initial message sent informing community pharmacists their patient has been admitted to hospital; then for these and consultation-referrals the referral waits for an electronic signal to be released. This occurs when the patient is discharged from hospital, when Refer-to-Pharmacy is automatically informed via a message from the hospital’s electronic patient administration system (PAS). Before sending a referral Refer-to-Pharmacy automatically checks that an electronic-discharge letter has been completed; if there is none, then the referrer and administrator are informed so that prompt action can be taken at ward level to ensure a letter is completed. This closes a potential loophole in a patient’s journey ensuring effective transfer of care to the GP too, who may otherwise be unaware their patient was in hospital let alone what medicines have changed. When a completed e-discharge letter is present *and* the patient has discharged the referral is automatically released with no further input required from the referrer.

## 4. Refer-to-Pharmacy—How It Works in Community Pharmacy

Referrals are held on the secure N3 network (*i.e.*, the NHS’s own computer network) and all messages are encrypted to meet information governance requirements. When a signal is received to release a referral (or to inform a community pharmacist that a care home or blister pack patient has been admitted to hospital) Refer-to-Pharmacy sends a prompting message to the community pharmacist to securely log in. The recipient can determine their preferred method of login prompt: fax notification, text, e-mail, or any combination of these. No patient information is provided at this stage, just the prompt to log in to access a referral. Refer-to-Pharmacy is web-based and does not use NHS-mail, which avoids the complications this can bring, particularly to some of the large multiple chains of community pharmacies that do not use NHS-mail.

Once securely logged in to the Refer-to-Pharmacy webpage, active referrals for that pharmacy are viewable. The pharmacist can see who has been referred and for what reason, then they either accept or reject the referral. They might reject a referral if they do not recognise a patient, although if a patient has intentionally chosen that pharmacy this can be made apparent via the referrer’s free-text comment. If a referral is unacknowledged within four days of receipt, a message is automatically sent back to the referrer and administrator so that the pharmacy can be telephoned to prompt them to log in and review their referral.

Once a referral is accepted, the community pharmacist can then view the patient’s full electronic-discharge letter. This is a copy of the complete letter sent to the GP including diagnosis, treatments, follow-up *etc.*, as well as information about changes to medicines, allergies, and the up to date list of prescribed medicines. For information-referrals the patient’s medication record at the pharmacy can be annotated with relevant information; for consultation-referrals, the pharmacist contacts the patient to arrange a mutually convenient time for them to visit the pharmacy for their consultation. The pharmacist may then prepare effectively for the consultation as they have a copy of the patient’s discharge letter.

Once a referral is completed, a process and/or clinical outcome is recorded from a dropdown menu to archive the referral. This also means Refer-to-Pharmacy can audit how many and of what type of referrals have been made from hospital, and what outcomes occurred. This makes it a useful tool for research, and also for benchmarking hospital referrers and (anonymously) community pharmacy outcome data.

**Figure 3:**
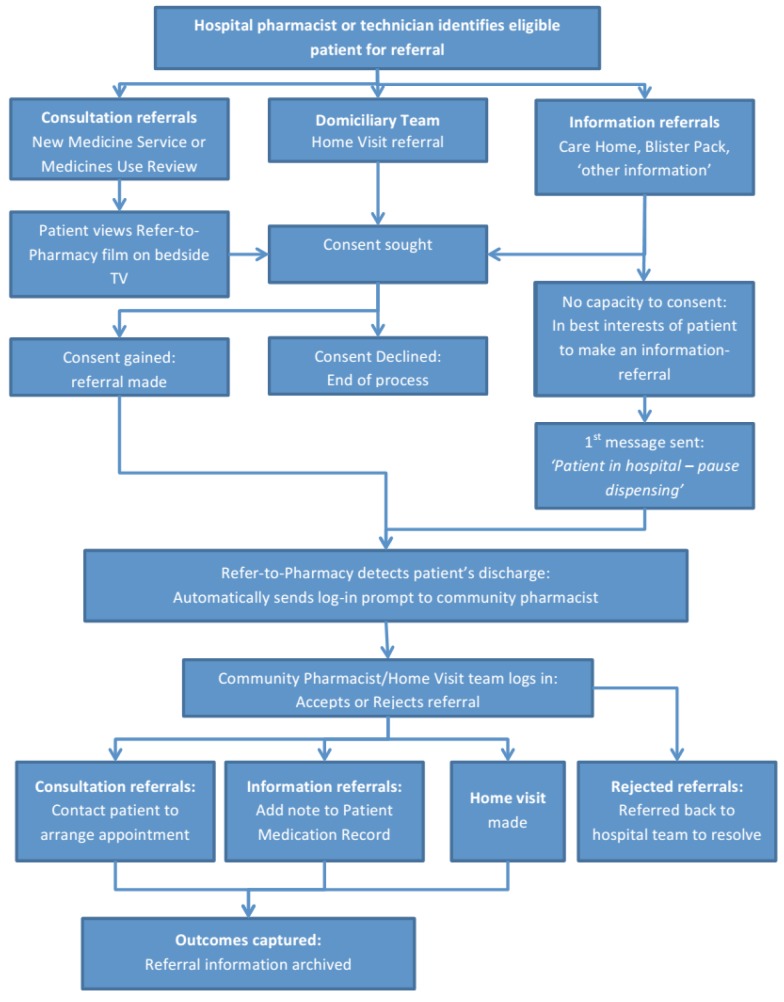
Refer-to-Pharmacy: overview flow diagram of referral process.

A flow chart of the whole process is shown in Figure 3.

## 5. Refer-to-Pharmacy in Your Health Economy

If you are considering introducing this solution, then a meeting of all key stakeholders is the starting point (hospital pharmacy lead, community pharmacy lead e.g., Local Pharmaceutical Committee (LPC) in England, clinical commissioner, hospital IT lead, and a representative from the software provider *i.e.*, Webstar-Health for Refer-to-Pharmacy). There is some cost associated with commissioning this kind of service and this is bespoke to any given health economy. Refer-to-Pharmacy has been designed to be spread to other health economies; it just needs the interfaces creating to allow communication with hospital information systems and the Refer-to-Pharmacy server, and this requires developer time. The cost of this can only be determined after a scoping meeting with key stakeholders.

The Royal Pharmaceutical Society has produced a toolkit to help spread referral systems of this type [9]. This gives people the arguments and facts for a case for change, and the practical steps needed to create a business case and to successfully implement a referrals solution.

## 6. Refer-to-Pharmacy and Medicines Optimisation

The strap line for Refer-to-Pharmacy is “get the best from your medicines and stay healthy at home” which in lay speech means “medicines optimisation”. In March 2015 the National Institute for Health and Care Excellence (NICE) published Medicines Optimisation guidance [10], which amongst other things states: “*a consenting person’s medicines discharge information should be shared with their nominated community pharmacy*”. Refer-to-Pharmacy provides the tool to make this possible, *and* to so routinely for *every* eligible patient.

Refer-to-Pharmacy is a tool to achieve an end, and relies on the training and knowledge of the pharmacy professionals at both ends of the process to deliver an effective service. This can be aided with a learning tool developed by the Centre for Pharmacy Postgraduate Education (CPPE) to help hospital teams identify eligible patients for referral, and for community teams to interpret discharge letters [11]. Other tools include the second edition of the Clinical Pharmacy Pocket Companion which has been designed to aid medicines optimisation in both hospital and community pharmacy in the knowledge that referrals will be made between the two [12].

## 7. Refer-to-Pharmacy and the Summary Care Record (SCR)

With moves now afoot to allow all community pharmacists to access patients’ SCRs [13], does this affect the Refer-to-Pharmacy concept? Not at all. If anything, it supports the need for pharmacists to access clinical information. An effective referral has to be timely; the community pharmacist needs to know when and what kind of action they need to take with a patient. The SCR greatly increases the information a community pharmacist can use, but it is only as accurate as the person owning the record makes it, and GPs have seven days to update their record of a patient post-discharge. Refer-to-Pharmacy provides an e-discharge letter with the latest medicines list at the same time that the GP receives it and *before* the SCR is updated. Crucially, community pharmacists are intentionally targeted by the hospital team to take specific, timely action with a patient—without a referral community pharmacists would never know a patient had discharged from hospital and was in need of their support.

## 8. Conclusions

Things really have changed in pharmacy and the pace of change is accelerating with improved information technology and professional leadership putting pharmacy at the forefront of patient care.

The next generation of pharmacists, whichever of the major branches of the profession they choose to start their professional lives in, should have the expectation that they will either refer patients to their community pharmacist, or receive referrals from hospital. Patients leaving hospital should expect….no, demand, to be referred. Refer-to-Pharmacy makes this possible.
